# Jefferson Medical College

**Published:** 1857-08

**Authors:** 


### JEFFERSON MEDICAL COLLEGE.

At a meeting of the Board of Trustees held on the 6th inst., Dr. Thos.

D. Mitchell of Philadelphia was elected to the chair of Materia Medica, and Therapeutics, made vacant by the resignation of Dr. R. M. Huston.

